# Prothrombotic Tendency in the Shadow of Cancer: Hypercoagulability, Impaired Clot Contraction and Fibrinolysis in Colorectal Cancer and Gastric Cancer Patients Undergoing Chemotherapy

**DOI:** 10.3390/ijms27042037

**Published:** 2026-02-21

**Authors:** Karolina Aleksandrowicz, Tomasz Rusak, Elżbieta Bołtromiuk, Tomasz Misztal, Barbara Polityńska-Lewko, Anna M. Wojtukiewicz, Joanna Kruszewska, Marta Myśliwiec, Marek Z. Wojtukiewicz

**Affiliations:** 1 Department of Population Medicine and Lifestyle Diseases Prevention, Medical University of Bialystok, Waszyngtona 15 B, 15-269 Bialystok, Poland; karolina.aleksandrowicz@sd.umb.edu.pl; 2 Bialystok Oncology Center, Ogrodowa 12, 15-022 Bialystok, Poland; 3 Department of Physical Chemistry, Medical University of Bialystok, Mickiewicza 2A, 15-222 Bialystok, Poland; elzbieta.boltromiuk@umb.edu.pl (E.B.); tomasz.misztal@umb.edu.pl (T.M.); 4 Department of Psychology and Philosophy, Medical University of Bialystok, Szpitalna 37, 15-295 Bialystok, Poland; barbara.politynska-lewko@umb.edu.pl (B.P.-L.); aniawojtukiewicz@gmail.com (A.M.W.); 5 Department of Oncology, Medical University of Bialystok, Ogrodowa 12, 15-269 Bialystok, Poland; joanna.kruszewska@umb.edu.pl (J.K.); marta.mysliwiec@umb.edu.pl (M.M.)

**Keywords:** hypercoagulability, clot contraction, fibrinolysis, cancer, chemotherapy

## Abstract

Cancer is frequently accompanied by thrombotic complications, but conventional coagulation tests often fail to adequately detect cancer-associated hypercoagulability. In this study, blood from 34 patients with colorectal or gastric cancers (before, during and after 3 months of chemotherapy) and 21 healthy controls was analyzed to obtain a coagulation–fibrinolysis profile (by thromboelastometry) and determine the kinetics of clot contraction (CCR). Patients demonstrated hypercoagulable profiles characterized by shortened clotting time, enhanced clot strength, reduced CCR, and impaired fibrinolysis compared to healthy controls, which was more pronounced in colorectal cancer patients. Parameters related to increased clot formation, impaired CC and delayed fibrinolysis correlated with disease stage. Chemotherapy meaningfully prolonged fibrinolysis, which was further evident in vitro with platinum-based drugs. Our findings reveal, for the first time, a significant inhibition of CCR in cancer patients. Integrating thromboelastometry with CCR measurements may improve stratification of cancer-associated thrombotic risk, which appears to arise from the hemostatic disturbances identified in our study. These may extend the duration of clot presence due to reduced fibrinolysis, while decreasing clot stability through diminished CCR. Therefore, careful monitoring of the precarious balance between thrombotic and bleeding tendencies may hold important implications for the fate of cancer patients.

## 1. Introduction

Cancer is frequently associated with profound disturbances of hemostasis, leading to thrombotic, hemorrhagic or thrombo-hemorrhagic complications. Venous thromboembolism (VTE), encompassing deep vein thrombosis and pulmonary embolism, represents the second leading cause of death among patients with cancer [1]. The risk of VTE is estimated to be four- to six-fold higher than in the general population and is further amplified by patient-specific characteristics, tumor biology, and therapeutic interventions such as surgery, radiotherapy, chemotherapy, immunotherapy and hormonal treatment [2,3]. Interestingly, the incidence of VTE differs among various cancer types. Cancers can be categorized into three major risk groups: high-risk (pancreatic, ovarian, gastric, hematologic, gynecologic cancers and central nervous system tumors), intermediate-risk (colorectal and lung cancers), and low-risk (breast and prostate cancers) [4,5]. The occurrence of VTE is an unfavorable prognostic factor, reducing overall survival and impairing treatment tolerance, further leading to treatment interruptions, dose reductions, or discontinuation of anticancer therapy, thereby indirectly affecting disease control and quality of life [4].

Conversely, hemorrhagic complications, affecting approximately 6–10% of patients with advanced cancer [6], pose an additional clinical challenge, further depicting the fragile imbalance of hemostasis in this population of patients. Thrombus formation within the vessel lumen restricts blood flow, activating compensatory mechanisms aimed at restoring vascular patency. Two tightly coordinated processes, clot contraction and fibrinolysis, are central to the flow restoration process by inducing a reduction in clot size and its subsequent dissolution [7]. Effective fibrinolysis and clot contraction aim to reduce the size of the developing clot to maintain blood flow. Clot contraction, mediated primarily by platelets, compacts fibrin networks and redistributes red blood cells, whereas fibrinolysis enzymatically degrades fibrin through plasmin activity. Dysfunction in either process may result in denser, more lysis-resistant, and thus long-lasting thrombi [7]. Unfortunately, detailed information on fibrinolytic disorders or clot contraction in cancer patients has been scarce and has not taken into account the interrelationship between them, therefore limiting the more complex evaluation of thrombotic risk in cancer patients. A better understanding of these relationships could help explain why some cancer patients develop persistent or recurrent thrombosis despite standard prophylaxis or therapeutic anticoagulation.

In patients with ischemic stroke [8] or venous thromboembolism [9], more obstructive clots form due to both weakened clot contraction and impaired fibrinolysis. The available information is somewhat scarce, as no “gold standard” test has yet been developed to assess fibrinolytic activity in the blood [10]. The available techniques are somewhat difficult to implement, time-consuming, and not sufficiently standardized, which complicates their comparison with other tests used to assess coagulation and fibrinolysis. Conventional plasma-based assays often fail to reflect the complex cellular contributions to clot dynamics, particularly the role of platelets and erythrocytes in whole-blood clot remodeling [11]. Furthermore, the detection of thrombotic events in cancer patients is mainly based on clinical symptoms, as conventional coagulation tests have limited effectiveness in detecting hypercoagulability. Rotational thromboelastometry (ROTEM) is an increasingly common hemostasis test used as a supportive tool during major surgery or trauma [12]. This test can detect many anomalies, from the initiation of clot formation through its propagation and stabilization up to its dissolution in the process of fibrinolysis. Recent research has shown that the use of thromboelastometry and thromboelastography yields accurate and efficient results in detecting blood hypercoagulability in advanced malignant tumors [13,14].

The present study aims to assess the relationships among clot formation, its stabilization, clot contraction and fibrinolysis in patients with colorectal and gastric cancers at diagnosis, during systemic therapy, and after three months of treatment. The effects of the administered treatment on alterations in hemostatic parameters in cancer patients were also evaluated. Focusing on these two gastrointestinal malignancies allows for comparison between intermediate- and high-risk cancer types for VTE, providing insight into cancer-specific hemostatic alterations over the course of treatment.

## 2. Results

Among the patients examined in the present study, men accounted for over 66% of the sample in both gastric and colorectal cancer, which is in keeping with the finding that these cancers are significantly more common in men than in women in clinical populations [15,16]. The vast majority (78%) of patients with colorectal cancer had stage IV disease (14/18) at the time of diagnosis, while among the patients with gastric cancer, those at stage IV accounted for 31% (5/16) of the sample. The demographic and clinical characteristics, as well as the median values of the parameters studied with the aid of thromboelastometry in all cancer patients and healthy controls, are summarized in Table 1.

As shown in Table 1, a slightly elevated platelet count in cancer patients and a significant increase in fibrinogen levels were present in cancer patients, in comparison to healthy control subjects. Thromboelastometric measurements showed a shortened clotting time and increased alpha angle, MCF and G compared to healthy individuals. These changes are indicative of hypercoagulability, which may be potentiated by impaired fibrinolysis. Indeed, both the lysis index (showing the percentage of clot strength relative to MCF at 45 and 90 min) and the lysis onset time (LOT) and total lysis time (LT) were significantly higher.

**Figure 1:**
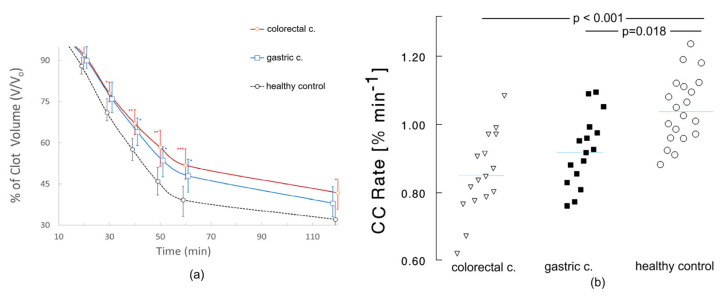
Progress of the clot contraction process in blood from a healthy subject and a cancer patient. The average time course of clot contraction in whole blood from healthy controls and colorectal or gastric patients are presented (**a**). The clot contraction rates based on clot volume measurements at specific time intervals; the clot retraction rates were calculated for each patient and control group and are presented in panel (**b**). The blue line marks the median. * *p* < 0.05; ** *p* < 0.01; *** *p* < 0.001.

Figure 1 shows that in comparison to healthy controls, cancer patients have a decreased clot contraction rate (CCR), both in the case of colon cancer and stomach cancer (0.840%·min^−1^ and 0.869%·min^−1^ vs. 1.015%·min^−1^). A significantly higher volume of clots at 40 min (64–67% vs. 58%, *p* < 0.05) in freshly collected whole blood of cancer patients was observed. There was no statistically significant difference in CCR and clot volumes between the colorectal and gastric cancers.

A comparison between the ROTEM parameters in cancer patients at different stages of illness (cancer stage, CS) demonstrated a shortened clotting time and an increase in alpha angle in CS IV patients, whereas a higher platelet count and clot strength were found in CS II/III patients. Additionally, fibrinolysis in CS II/III was slower, which may be due not only to greater cross-linking of the clot (suggested by the elevated G parameter), but also to profoundly altered fibrinolysis in CS IV. In the analyzed CS IV group, we observed impaired fibrynolysis (hipofibrynolysis) in only six patients, whereas the remaining twelve patients exhibited clear hyperfibrinolysis. Correspondingly, the results presented in Table 2 indicated that the lysis onset time and total fibrinolysis were shorter in the more advanced cancer stages.

Cancer patients have a higher risk of thromboembolic complications in comparison to the healthy population [1,2,3,4,5], a finding which was also confirmed by our observations. On completion of the study, between 5 and 9 months after the first examination, six patients had developed a pulmonary embolism, seven deep vein thrombosis (including two patients who had both conditions), and two experienced major bleeding. Some patients had a port device (an additional risk factor), three of whom had a visible clot at the tip of the vascular catheter. In most of these patients, the tests showed hypercoagulability (mean CT = 242 s; α = 70°, G = 11.62 kdyn/cm^2^), reduced clot contraction (CRR = 0.874%·min^−1^), or delayed fibrinolysis (LOT = 62 min; LT = 158 min). Among the analyzed parameters, the most pronounced changes suggesting a developing hypercoagulable state were observed in G, LT and CRR. In the patients who later developed bleeding, fibrinolysis times were significantly shorter (LT ~ 90 min) and were further shortened during treatment.

**Figure 2:**
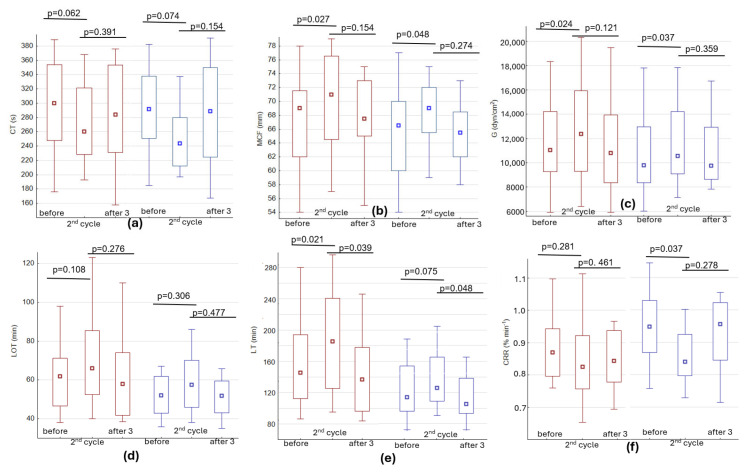
Changes in ROTEM parameters and clot contraction rate in colorectal (marked in red) and gastric (marked in blue) patients during chemotherapy indicating altered hypercoagulability: (**a**) clotting time results (changes during clotting initiation); (**b**,**c**) the stabilization of the developing clot (maximum clot firmness and clot strength, respectively); (**d**) lysis onset time (LOT); (**e**) complete lysis time (LT); (**f**) changes in the rate of clot contraction (CCR). Median values with interquartile range (1st–3rd, frame box) and range (min-max, whiskers) are presented. The analyzed groups were compared with the aid of Wilcoxon’s paired test.

**Figure 3:**
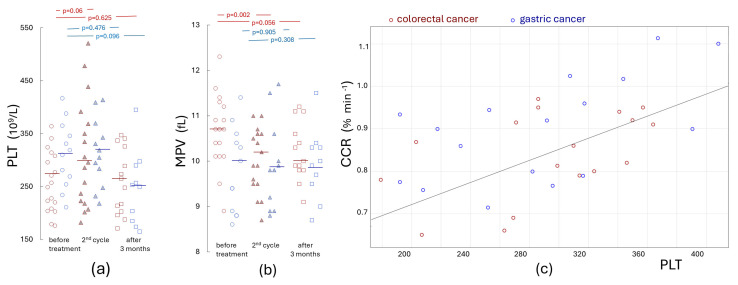
Changes in platelet count (PLT, (**a**)) and MPV (**b**) in patients with colorectal and gastric cancer undergoing chemotherapy. Red markers indicate colorectal cancer patients; blue markers indicate gastric cancer patients. Unfilled circles represent patients before treatment, triangles before the second cycle, and squares after 3 months of therapy. Horizontal bars represent median values. The analyzed groups were compared with the aid of Wilcoxon’s paired test. The correlation of clot contraction rate with platelet count in cancer patients is presented in panel (**c**).

Since chemotherapy increases the risk of thrombosis [17,18], we decided to assess whether changes in hypercoagulability occur during treatment. Patients with colorectal cancer were treated with chemotherapy, consisting of 5-fluorouracil and folinic acid (18/18) in addition to which irinotecan (16/18) or oxaliplatin (2/18) were also used. In patients with gastric cancer, 5-fluorouracil was equally the treatment of choice (13/16), in combination with platinum-based agents (12/16) and docetaxel (5/16) or irinotecan (2/16). Figure 2 shows that some coagulation and fibrinolysis parameters were altered in the direction of increased coagulability during treatment. Although changes related to clot formation were only slightly altered (statistically insignificant, Figure 2a), clot amplitude (Figure 2b) and strength Figure 2c,d) increased significantly, and fibrinolysis was clearly slower. This was most evident in the time required for complete clot lysis (LT—measured as a 90% reduction in MCF, Figure 2e). It was found that the average G increased by 1251 dyn/cm^2^ and average lysis time increased by 22 min in colon cancer, and in gastric cancer by 753 dyn/cm^2^ and 8 min, respectively. Clot contraction rates also decreased after chemotherapy (0.869 vs. 0.828; *p* = 0.281 and 0.947 vs. 0.840%·min^−1^; *p* = 0.037 in colorectal and gastric cancer, respectively) although in some cases, especially in those with an increase in platelet count, a marked acceleration in clot contraction was observed. The CCR in cancer patients strongly correlated with platelet count (R = 0.589; *p* < 0.001, shown in Figure 3c), clot strength (R = 0.539; *p* < 0.005), LOT (R = 0.594; *p* < 0.005) and LT (R = 0.49; *p* < 0.05). As shown in Figure 3a, a slight increase in platelet count was observed in cancer patients (mainly in colorectal) before the second cycle of chemotherapy, followed by a slight decrease after 3 months. Despite increased platelet counts in patients with colorectal cancer, a slight decrease in mean platelet volume (MPV) was observed before the second cycle of chemotherapy. After three months, changes in platelet counts and hypercoagulability parameters tended to return to values similar to those observed at the beginning of the study, while reduced MPV values remained at a slightly lower level.

**Figure 4:**
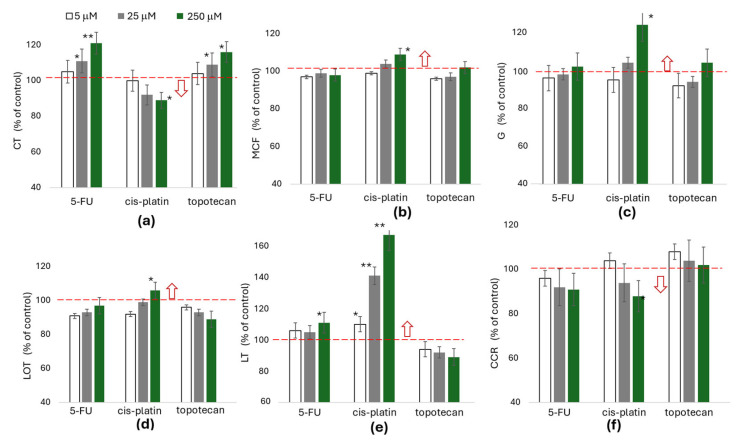
The effect of 5-fluorouracil (5-FU), cisplatin and topotecan on ROTEM parameters and CCR in platelet-rich plasma of healthy volunteers. PRP samples after preincubation (10 min at ambient temperature) with the indicated concentrations of the drugs under investigation (except for the control group, which was drug-free). Samples analyzed with regard to the kinetics of clot formation, stabilization and fibrinolysis using rotational thromboelastometry (**a**–**f**). The kinetics of the clot contraction rate (CCR) were evaluated based on images of contracted clots, which were photographed every 10 min. The pictures were processed in dedicated software to obtain the percentage of contraction at the indicated time intervals. Clot contraction rates (for each sample) were further calculated from these data. Median values and interquartile range (1st–3rd) are presented. N = 14, * *p* < 0.05, ** *p* < 0.005 (compared to control expresses as 100%). Red arrow indicates which direction of changes is associated with hypercoagulability, while the red line indicates the 100% value as a reference point (control).

To assess whether chemotherapy may augment hypercoagulability, we further investigate the effects of 5-fluorouracil, cisplatin, and topotecan (a topoisomerase I inhibitor) on the kinetics of clot formation (Figure 4a–c) and fibrinolysis (Figure 4d,e), in addition to the kinetics of clot contraction (Figure 4f). In order to address the questions raised in relation to chemotherapy we used platelet-rich plasma (PRP), which allowed for the assessment of the direct effect of the drugs concerned on platelets, whilst excluding other blood morphological elements, in particular erythrocytes—the most numerous morphotic elements of blood. A bolus addition of the studied compounds was used at concentrations corresponding to the maximum therapeutic doses [19], as well as at concentrations 5–10-fold or 50–100-fold higher. The obtained results were expressed as a percentage change compared to the control sample (who did not demonstrate drug dependency), which was assigned a value of 100% for each comparison. 5-fluorouracil and topotecan, regardless of the concentration used, did not significantly affect coagulation parameters or clot contraction kinetics. However, cisplatin, especially at concentrations exceeding single therapeutic doses, showed a slight enhancement of coagulation processes and inhibition of clot contraction and fibrinolysis.

## 3. Discussion

Thromboembolic complications occur much more frequently in cancer patients than in healthy individuals, but the detection of hypercoagulability in this patient group has been constrained by a methodological issues. The results of this study indicate that the use of ROTEM may help improve the detection of coagulation disorders. The hypercoagulable state observed in most patients is usually characterized by a shortened clotting time (CT), a shortened clot formation time (CFT), an increase in the alpha angle, and an increase in MCF and G [20]. Comparison of CT and CFT between patient groups showed a slight and non-significant reduction, which may explain the low effectiveness of conventional coagulation tests in detecting hypercoagulability. The clotting time provides information about clot activation and initiation, whereas CFT measures the propagation phase of coagulation, i.e., specifically how fast the fibrin clot strengthens after initiation, thus reflecting thrombin generation. Although a significant acceleration of the initial phase of coagulation was observed in gastric cancer, these changes were less evident in patients with colorectal cancer (Table 1). Much more noticeable changes indicating hypercoagulability in this group were observed in the MCF and its G. These results are in agreement with other reports, where often the maximal amplitude of the clot has been characterized as the most useful parameter in the assessment of hypercoagulability using the thromboelastography (TEG) method [14,21].

It is known that both fibrinogen and blood platelets significantly affect clot development and its strength, and their increased levels contribute to the hypercoagulable state. In healthy individuals, fibrinogen contributes functionally to approximately 20% of clot strength, while platelets contribute to 80% [22]. In fact, cancer patients had higher platelet counts, but these differences were not statistically significant in comparison to healthy controls. However, considering that cancer cells can trigger platelet activation [23] and taking into account the elevated fibrinogen levels, this may at least partially explain the increased amplitude and strength of the formed clots in the cancer groups examined. It is highly likely that platelet-related thrombin production and fibrinogen availability lead to the formation of greatly crosslinked platelet–fibrin clots that are more resistant to lysis [7,24]. Indeed, as we have shown here, both LOT and LT and fibrinolysis progression at 45–90 min (LI45–LI90) were significantly impaired, which, in combination with the existing state of hypercoagulability, would be expected to contribute to the increased risk of developing deep vein thrombosis and pulmonary embolisms in cancer patients [25]. Notably, some patients experienced thrombotic events during the course of our study, which required initiation of antithrombotic therapy. These observations highlight the potential value of methods for early detection of increased thrombotic risk, while current guidelines still do not recommend routine thromboprophylaxis in the absence of clinical complications. Our observations suggest that the development of new methods for assessing hypercoagulability status may be a useful tool for developing more personalized antithrombotic prophylaxis, even before the onset of clinical symptoms.

Fibrinolysis is often disturbed in cancer patients, where both hypercoagulation and accelerated clot breakdown (hyperfibrinolysis), as well as a slowed lysis process, may occur [26]. Such observations were particularly evident in the group of patients with stage IV metastatic disease, where increased fibrinolytic activity was frequently observed. This can significantly complicate the definitive assessment of hypercoagulability, because excessively formed blood clots can also be disrupted faster. In this context, the assessment of the kinetics of clot contraction may provide additional information.

The results presented here show, for the first time, that cancer is associated with a significant inhibition of clot contraction, which is crucial for stabilizing the developing clot and preventing thrombosis [7,27]. We also demonstrated a strong correlation between platelet count and clot contraction rate in our study. Inhibition of clot contraction was more pronounced in patients with colorectal cancer, among whom the percentage of patients with elevated platelet counts was lower. Considering that clinical conditions characterized by thrombocytosis are characterized by an increased CRR, the obtained results are not surprising. Additionally, data analysis also showed that after excluding patients with platelet counts exceeding 300 × 10^9^/L, the differences in CRR between patients with colorectal and gastric cancer decreased significantly, reaching similar values of 0.836 and 0.872%·min^−1^, respectively—indicating even greater inhibition of clot contraction in cancer compared to the control group.

Assessment of global hemostasis in cancer patients indicates that patients with colorectal cancer may be at increased risk of thrombotic complications compared to gastric cancer. In studies using the ROTEM method it has been observed that, whilst the initiation of the coagulation process is only affected to a minor extent, the clots formed in colorectal cancer are characterized by greater MCF and G, as well as increased resistance to contraction and fibrinolysis. These results are consistent with previous reports, which indicate that colorectal cancer is associated with a higher risk of VTE compared to gastric cancer (classified as intermediate risk of thrombosis) [4,5,28]. The convergence of our observations with data from the literature supports the hypothesis that patients with colorectal cancer might be particularly predisposed to prothrombotic hemostasis disorders. One reason for this may be the significant impact of the architecture of the clot on the kinetics of its lysis and contraction [29,30,31]. In general, highly crosslinked fibrin strands (denser clots) are stiffer, less elastic, and more resistant to breakdown (fibrinolysis) and clot contraction, which could contribute to hemostatic dysregulation in cancer. Less compacted or poorly contracted thrombi significantly impair blood flow and are highly prone to rupture and to increasing the risk of embolization [8,9,32]. Additionally, impaired clot contraction signals platelet dysfunction and a related reduction in platelet contractility, which may indicate a risk of bleeding [33,34,35]. Some individuals with symptoms of chronic bleeding have been found to have platelet subpopulations with diminished platelet contractile forces [35]. It is possible that inhibition of clot contraction could explain the two cases of bleeding experienced by the two patients in our study mentioned earlier. Therefore, clot contraction disorders may contribute to both thrombotic and hemorrhagic complications. An additional factor contributing to the development of thrombosis is chemotherapy. The use of potent drugs to treat cancer has been associated with a two- to six-fold increase in the risk of VTE [17,18]. As shown in Figure 2, the significance of increased hypercoagulability was observed before the second cycle of chemotherapy, which may be due to the slight increase in platelet count, a decrease in MPV, and the drugs used during treatment. Assessing whether the medications employed affected hypercoagulability was complicated due to the wide range of agents used. In order to assess whether the drugs could potentially affect the kinetics of coagulation, contraction or fibrinolysis, their possible influence at concentrations exceeding therapeutic doses on selected parameters recorded in healthy individuals in vitro was analyzed. Although single doses of fluorouracil or a topoisomerase inhibitor had no effect on increases in the parameters indicating a hypercoagulable state, an effect of this kind was evident with the use of a supratherapeutic concentration of platinum-based agents. Considering that the effects of some drugs can be cumulative, their total effect on the impairment of hemostasis cannot be ruled out. It also cannot be excluded that the use of chemotherapeutic agents leads to a reduction in MPV [36], which may consequently result in diminished platelet activity, including their crucial role in clot contraction. Accordingly, the observed impairment of clot contraction before the second cycle of chemotherapy may be consistent with this mechanism.

We recognize that our study has a number of limitations, mainly related to the heterogeneity of the cohorts and their relatively small size. Our study was designed as a pilot investigation and certainly requires validation and confirmation of the obtained results in future studies, using other methods such as thrombin generation assay (TGA) or specific blood biomarkers, such thrombin–antithrombin complexes and prothrombin fragment 1 + 2 (F_1+2_). TGA measures the ability of plasma to generate thrombi, including initiation, propagation, and endogenous thrombin potential, while remaining sensitive to impaired inhibitory mechanisms and circulating procoagulant microparticles released by tumor cells [37,38]. TGA is excellent for assessing hypercoagulability and could be complementary to thromboelastometry, especially in view of the fact that some parameters of ROTEM values display a remarkable degree of similarity to endogenous thrombin potential data.

We chose to use thromboelastometry (ROTEM) because it allows for the assessment of the viscoelastic properties of whole blood (clotting time, speed, and strength), encompassing the kinetics of clot formation, clot growth, and susceptibility to fibrinolysis. TGA, on the other hand, measures the ability of plasma to generate thrombin, but in an environment where erythrocytes—the most abundant morphological elements in blood—are absent.

The wide range of available treatment options is an additional complication in reliably assessing coagulation disorders in cancer patients. Other authors have previously shown that thromboelastography can be used to assess hypercoagulation in tumors [13,39], but we have demonstrated for the first time that impaired clot contraction may also contribute to an increased risk of thrombosis in these patients. It is important to assess all stages of hemostasis, from clot formation through its stabilization and susceptibility to contraction and fibrinolysis.

Most importantly, our findings suggest that subclinical activation of coagulation accompanied by impaired fibrinolysis, indicative of a prothrombotic tendency, may represent a subtle process that remains concealed during the course of malignancy. Such hemostatic disturbances contribute to an increased risk of thromboembolic complications in cancer patients. Notably, patients with colorectal and gastric cancer in the present study also exhibited impaired clot contraction, which may indicate an elevated risk of bleeding. These observations highlight the importance of careful monitoring of the delicate balance between prothrombotic and bleeding tendencies in this population. The presented results may have meaningful clinical implications and support a personalized approach to antithrombotic prophylaxis in cancer patients, potentially improving clinical outcomes.

## 4. Materials and Methods

### 4.1. Patients

A total of 125 blood samples were used in this study, taken from fifty-five adults (21 healthy individuals and 34 cancer patients). We included 18 patients with colorectal cancer (6 women, 12 men) and 16 with gastric cancer (5 women, 11 men) aged 44 to 74 years. Patients with thrombocytosis or thrombocytopenia, pre-existing coagulation disorders (such as thrombosis or hemophilia), anticoagulant therapy, or significant comorbidities that could affect coagulation parameters were excluded from the study. A sex- and age-matched control group of 21 healthy volunteers (7 women, 14 men) was also studied.

### 4.2. Blood Collection and Preparation

Venous blood was collected with minimum trauma and stasis via a 21-gauge needle (0.8 × 40 mm) into 10 mL polypropylene tubes containing 1 mL of 130 mM (3.2%) trisodium citrate. Blood samples were collected before treatment, before the second cycle of chemotherapy and three months after starting treatment. All procedures were conducted in accordance with the principles of the World Medical Association Declaration of Helsinki, and the study was approved by the local Ethics Committee on human research (RI-I-002/558/2019; date of consent: 15 December 2022). The privacy rights of human subjects were observed and written informed consent was obtained from all the blood donors. Platelet-rich plasma (PRP) was obtained by centrifugation of whole blood at 200× *g* for 20 min. To prepare platelet-poor plasma (PPP), PRP was centrifuged at 2800× *g* for 10 min.

### 4.3. Thromboelastometric Measurements

Kinetics of whole-blood thrombus formation and fibrinolysis were performed using the ROTEM^®^ Delta rotational thromboelastometric system (Tem International GmbH, Munchen, Germany). All ROTEM coagulation measurements were performed by the same experienced operator as follows: 320 μL of blood was transferred into a preheated (37 °C) cup, containing 20 μL of 0.2 M calcium chloride, and repeatedly pipetted gently to mix the components [40]. To evaluate the coagulation kinetics, we measured the following parameters: clotting time—time from start of measurement to the beginning of the fibrin polymerization process; clot formation time (CFT)—time to reach 20 mm of clot amplitude; alpha angle (α)—the angle showing the dynamics of clot formation; maximum clot firmness (MCF)—a parameter reflecting the strength of the formed clot to resist the pin oscillation; and shear elastic modulus strength (G). All of the above mentioned variables may be described as follows: clotting time and CFT as the variables indicating clot formation; α as parameters reflecting clot propagation; and MCF and G as the variables displaying clot stabilization. Fibrinolytic potential was assessed in recalcified blood by either 140 ng/mL tissue factor (TF) and 125 ng/mL of tissue plasminogen activator (tPA) [40,41], and parameters including Lysis Index (LI), quantifying the percentage of clot strength remaining at specific times (e.g., LI30, LI60); or by measuring the time after which the clot amplitude decreased by 15% (LOT—lysis onset time) and 90% (LT—lysis time) relative to MCF and analyzing the results [12,20].

### 4.4. Measurements of Kinetics of Clot Contraction

Measurement of the kinetics of clot contraction in whole blood was performed in non-siliconized glass tubes as described previously [42]. Pictures were taken for one hour at 10 min intervals and after 120 min using a digital camera. Quantification of contraction was performed by assessment of the clot area using Motic Images Plus 2.0 ML software, and data were processed using Microsoft Excel. Clot surface areas were plotted as a percentage of maximal contraction (i.e., volume of platelet suspension). Data were expressed as follows: percentage of contraction (relative clot volume) = (area t_0_ − area t)/(area t_0_) × 100. The kinetics of clot contraction was characterized by the calculation of the rate constant of the contraction process (CCR).

### 4.5. Routine Hematological Assays

The complete blood cell count was measured by an automated hematology analyzer (Sysmex SE9000, Toa Medical Electronics, Kobe, Japan), and the fibrinogen plasma level was determined by the Clauss Method using a coagulation analyzer Coag-Chrome 3003 (Bio-ksel, Grudziadz, Poland).

### 4.6. Data Analyses

Data were evaluated using STATISTICA 14.1.0.4 software (StatSoft, Tulsa, OK, USA). Differences between the groups were assessed with the aid of the Mann–Whitney U test. The data are shown as the median (range minimum–maximum) of the number of determinations (n) or as a percentage compared to the control. In all experiments, a *p* value of <0.05 was considered to be significant. Correlations were assessed by a non-parametric test (Spearman’s rank correlation coefficient, r).

## 5. Conclusions

Our findings reveal, for the first time, a significant inhibition of clot contraction in cancer patients. It would seem that the increased thromboembolic risk in cancer patients may arise from such subclinical hemostatic disturbances, characterized by coagulation activation and impaired fibrinolysis, which remain obscured during the course of malignancy. We suggest that careful monitoring of the balance between thrombosis and bleeding is therefore crucial, and further research and a better understanding of clot remodeling may contribute to the development of more personalized and safer thromboprophylaxis in cancer patients.

## Figures and Tables

**Table 1:** Characteristics of healthy control subjects and patients with cancer—the dynamics of coagulation and fibrinolysis in ROTEM measurements.

	Colorectal Cancer (*n* = 18)	Gastric Cancer (*n* = 16)	Healthy Control (*n* = 21)
Age	61 (44 ÷ 73)	62 (49 ÷ 73)	61 (45 ÷ 74)
Sex (F/M)	6/12 (33%/67%)	5/11 (31%/69%)	7/14 (33%/67%)
PLT (10^9^/L)	269 (173 ÷ 364)	283 (176 ÷ 417)	248 (159 ÷ 354)
Fibrinogen (g/L)	3.8 * (2.86 ÷ 5.62)	3.94 * (2.51 ÷ 5.11)	3.04 (2.42 ÷ 4.3)
CT (s)	296 (166 ÷ 400)	288 * (224 ÷ 436)	327 (217 ÷ 421)
CFT (s)	122 (61 ÷ 171)	108 * (76 ÷ 172)	131 (71 ÷ 159)
α (°)	68 (60 ÷ 74)	71 * (61 ÷ 76)	66 (56 ÷ 71)
MCF (mm)	69 * (54 ÷ 79)	66 * (54 ÷ 78)	63 (54 ÷ 71)
G (kdyn/cm^2^)	11.03 ** (5.91÷18.34)	9.88 * (6.0÷17.81)	8.06 (5.73÷12.47)
LOT (min)	62 * (41 ÷ 98)	53 (35 ÷ 72)	43 (30 ÷ 45)
LI45 (%)	94 * (81 ÷ 99)	93 * (74 ÷ 99)	84 (48 ÷ 92)
LI90 (%)	53 ** (12 ÷ 88)	36 ** (12 ÷ 76)	0 (0 ÷ 28)
LT (min)	143 ** (98 ÷ 300)	113 * (58 ÷ 188)	74 (52 ÷ 114)

PLT—platelet number, CT—clotting time, CFT—clot formation time, α—slope of line during clot formation, MCF—maximum clot firmness (amplitude), G—clot strength (shear modulus), LOT—lysis onset time, LI45, LI90—lysis index at 45, 90 min, LT—lysis time. Medians with range (min ÷ max) are presented. Changes indicating hypercoagulability are marked in red. * *p* < 0.05; ** *p* < 0.01 (in comparison to control).

**Table 2:** Comparison of the parameters analyzed in patients at CS II/III and CSIV of illness.

	CS IV (*n* = 19)	CS II/III (*n* = 15)
age	62 (44 ÷ 74)	60 (50 ÷ 74)
PLT (10^9^/L)	279 (173 ÷ 470)	325 (204 ÷ 471)
fibrinogen (g/L)	3.87 (2.86 ÷ 5.62)	3.98 (2.51 ÷ 5.59)
CT (s)	294 (175 ÷ 436)	323 (185 ÷ 390)
CFT (s)	117 (71 ÷ 172)	116 (86 ÷ 152)
α (°)	71 (60 ÷ 78)	68 (61 ÷ 75)
MCF (mm)	67 (54 ÷ 76)	67 (61 ÷ 78)
G (kdyn/cm^2^)	10.18 (5.91–14.35)	10.85 (7.76–18.34)
LOT (min)	51 (35 ÷ 77)	60 (51 ÷ 98)
LI45 (%)	90 (74 ÷ 99)	97 (93 ÷ 99)
LI60 (%)	70 (4 ÷ 96)	90 (79 ÷ 95)
LI90 (%)	36 (12 ÷ 76)	66 (20 ÷ 88)
LT (min)	90 (56 ÷ 178)	129 (104 ÷ 267)
CV40 (%)	64 (50 ÷ 77)	62 (49 ÷ 69)
CV60 (%)	49 (36 ÷ 62)	44 (33 ÷ 54)
CCR (%·min^−1^)	0.876 (0.61 ÷ 1.08)	0.952 (0.81 ÷ 1.14)

CV40, CV60—clot volume at 40 or 60 min, CCR—clot contraction rate. Medians with range (min ÷ max) are presented. Changes that may indicate increased hypercoagulability are marked in red. No significant statistical differences were found between the groups.

## Data Availability

Requests for data will be honored on a case-by-case basis, and data will be provided by the corresponding authors.

## Abbreviations

The following abbreviations are used in this manuscript:

CRR	Clot Contraction Rate
CFT	Clot Formation Time
CS	Cancer Stage
CT	Clotting Time
5-FU	5-fluorouracil
G	shear elastic modulus strength (clot strength)
LI	Lysis Index
LOT	Lysis Onset Time
LT	(complete) Lysis Time
MCF	Maximum Clot Firmness (maximum amplitude of clot)
PRP	Platelet-Rich Plasma
ROTEM	Rotational Thromboelastometry
TGA	Thrombin Generation Assay
TEG	Thromboelastography
TF	Tissue factor
tPA	tissue Plasminogen Activator
VTE	venous thromboembolism
